# Ultra high dose rate (35 Gy/sec) radiation does not spare the normal tissue in cardiac and splenic models of lymphopenia and gastrointestinal syndrome

**DOI:** 10.1038/s41598-019-53562-y

**Published:** 2019-11-20

**Authors:** Bhanu Prasad Venkatesulu, Amrish Sharma, Julianne M. Pollard-Larkin, Ramaswamy Sadagopan, Jessica Symons, Shinya Neri, Pankaj K. Singh, Ramesh Tailor, Steven H. Lin, Sunil Krishnan

**Affiliations:** 1 Departments of Experimental Radiation Oncology, University of Texas MD Anderson Cancer Center, Houston, Texas USA; 2 Radiation Oncology, University of Texas MD Anderson Cancer Center, Houston, Texas USA; 3 Department of Radiation Physics, University of Texas MD Anderson Cancer Center, Houston, Texas USA; 4 The University of Texas MD Anderson Cancer Center-UT Health Graduate School of Biomedical Sciences, Houston, Texas USA; 5 Department of Radiation Oncology, Mayo Clinic Florida, 4500 San Pablo Road S, Jacksonville, FL 32224 USA

**Keywords:** Pancreatic cancer, Translational research, Biological physics

## Abstract

Recent reports have shown that very high dose rate radiation (35–100 Gy/second) referred to as FLASH tends to spare the normal tissues while retaining the therapeutic effect on tumor. We undertook a series of experiments to assess if ultra-high dose rate of 35 Gy/second can spare the immune system in models of radiation induced lymphopenia. We compared the tumoricidal potency of ultra-high dose rate and conventional dose rate radiation using a classical clonogenic assay in murine pancreatic cancer cell lines. We also assessed the lymphocyte sparing potential in cardiac and splenic irradiation models of lymphopenia and assessed the severity of radiation-induced gastrointestinal toxicity triggered by the two dose rate regimes *in vivo*. Ultra-high dose rate irradiation more potently induces clonogenic cell death than conventional dose rate irradiation with a dose enhancement factor at 10% survival (DEF_10_) of 1.310 and 1.365 for KPC and Panc02 cell lines, respectively. Ultra-high dose rate was equally potent in depleting CD3, CD4, CD8, and CD19 lymphocyte populations in both cardiac and splenic irradiation models of lymphopenia. Radiation-induced gastrointestinal toxicity was more pronounced and mouse survival (7 days *vs*. 15 days, *p* = 0.0001) was inferior in the ultra-high dose rate arm compared to conventional dose rate arm. These results suggest that, contrary to published data in other models of radiation-induced acute and chronic toxicity, dose rates of 35 Gy/s do not protect mice from the detrimental side effects of irradiation in our models of cardiac and splenic radiation-induced lymphopenia or gastrointestinal mucosal injury.

## Introduction

Radiation therapy (RT) is an integral component of cancer treatment with at least 50% of cancer patients receiving definitive or palliative RT sometime during the course of their cancer treatment^1^. Advancements in treatment delivery in the last three decades has seen the increasing utilization of sophisticated techniques such as three-dimensional conformal RT, intensity modulated RT, imaged guided RT, stereotactic RT, and charged particle therapy. All of these techniques allow greater precision with physically sculpting radiation dose to the tumor while minimizing dose to adjacent normal tissues, yet the ability to dramatically increase tumor dose while respecting normal tissue tolerance has only changed incrementally. Favaudon *et al*. recently reported an entirely new paradigm that has the potential to dramatically broaden the therapeutic window allowing substantial dose escalation to the tumor and/or considerable reduction in acute and late toxicity from incidental normal tissue irradiation. This approach used ultra-high dose rate electron irradiation (4.5 MeV electrons at ≥40 Gy/s, termed FLASH radiation). FLASH caused less acute and late radiation-induced lung injury (pneumonitis and fibrosis, respectively) and upregulation of transforming growth factor-beta (TGF-beta), a biomarker of radiation fibrosis, than conventional dose rate RT. They also demonstrated that FLASH RT caused less acute capillary endothelial apoptosis, an early marker of normal organ injury, in mouse lungs than conventional dose rate RT. Importantly, however, FLASH RT was no less effective than conventional dose rate RT in arresting or inhibiting tumor growth in heterotopic or orthotopic breast and lung cancer models^2^. Though the mechanistic underpinnings of this remarkable phenomenon remain largely unclear, this normal tissue sparing property of FLASH RT has now been reproduced in a number of preclinical models of normal tissue injury and cancer control^2–5^. In the clinical realm, current technologies and treatment geometries do not have the capability to achieve these high dose rates with photons - flattening filter-free linear accelerators achieve dose rates as high as 0.4 Gy/s compared to conventional dose rates of 1–4 Gy/min^6^. Cyclotron-based proton irradiators can, however, achieve dose rates as high as 200 Gy/s^7^. And novel accelerator designs can, in principle, irradiate tumors with FLASH-range very high energy electrons or photons.

We explored the likelihood that FLASH-range dose rates may spare radiation-induced lymphopenia, a relatively common adverse effect of RT that significantly reduces tumor control and patient survival in a number of primary tumor irradiation scenarios. Lymphocytes, among the most radiosensitive cells in the body, are routinely subject to unintended radiation while circulating through the vasculature within the tumor and surrounding tissues within the irradiation portal, residing in lymph nodes and secondary lymphoid organs in the treatment field, and/or lodged in the heart that may be within the treatment field. Unintentional irradiation of these lymphocyte compartments has been implicated in radiation-induced lymphopenia (RIL), which in turn, has been increasingly recognized as an independent predictor of inferior overall survival (OS) in gliomas, pancreatic cancer, lung cancer, and hepatocellular carcinoma, and inferior progression free survival (PFS) in esophageal cancer and head and neck cancers^8–13^. RIL has also been associated with poor response to therapy and higher recurrence rates in cervical and bladder cancer^14,15^. Circulating lymphocyte count is also an important biomarker of response to immunotherapeutic agents like immune checkpoint inhibitors^16^. As clinical trials attempt to combine RT with immune checkpoint inhibitors, sparing the lymphocyte populations may be especially germane to the ability to maximizing the systemic immune response and augmenting distant tumor control.

Whereas most studies demonstrate RIL as a poor prognostic factor in cancer treatment outcomes, the direct correlation between dose to lymphocyte-rich organs and RIL has been most recently described in pancreatic cancer patients receiving unintentional splenic RT and esophageal cancer patients receiving unintentional cardiac and splenic RT^10,17,18^. Using irradiation of these two lymphocyte-rich organs as our model system for RIL, we studied the potential normal tissue sparing ability of ultra-high dose rate RT. We surmised that the benefit of such a technique of RIL reduction could impact a wide range of clinical scenarios including treatment of esophageal, lung, breast, pancreatic and hepatobiliary cancer patients, where RIL is associated with inferior OS and/or reduced complete pathological response rates^11,19–22^.

## Results

### Dosimetric verification of dose and dose rate

We utilized Gafchromic film to confirm each dose delivery in either setup and also used a CC04 Farmer chamber for dose confirmation in our FLASH system. The reproducibility of delivered dose varied within each session of our FLASH irradiation. In order to improve reproducibility, we would perform a daily warm up of 1000 MU on the FLASH linear accelerator and give a 6 sec delay of the gating signal which resulted in 1.5% output variation. A warm up of 1000 MU and gating delays from 3–9 seconds improved reproducibility. We measured a standard deviation of delivered dose for a gate of 1 second was about 1% open field and about 2% for a 2 × 2 cm^2^ field at the level of the mirror.

### Ultra-high dose rate RT causes more apoptosis and clonogenic cell death than conventional dose rate RT

**Figure 1:**
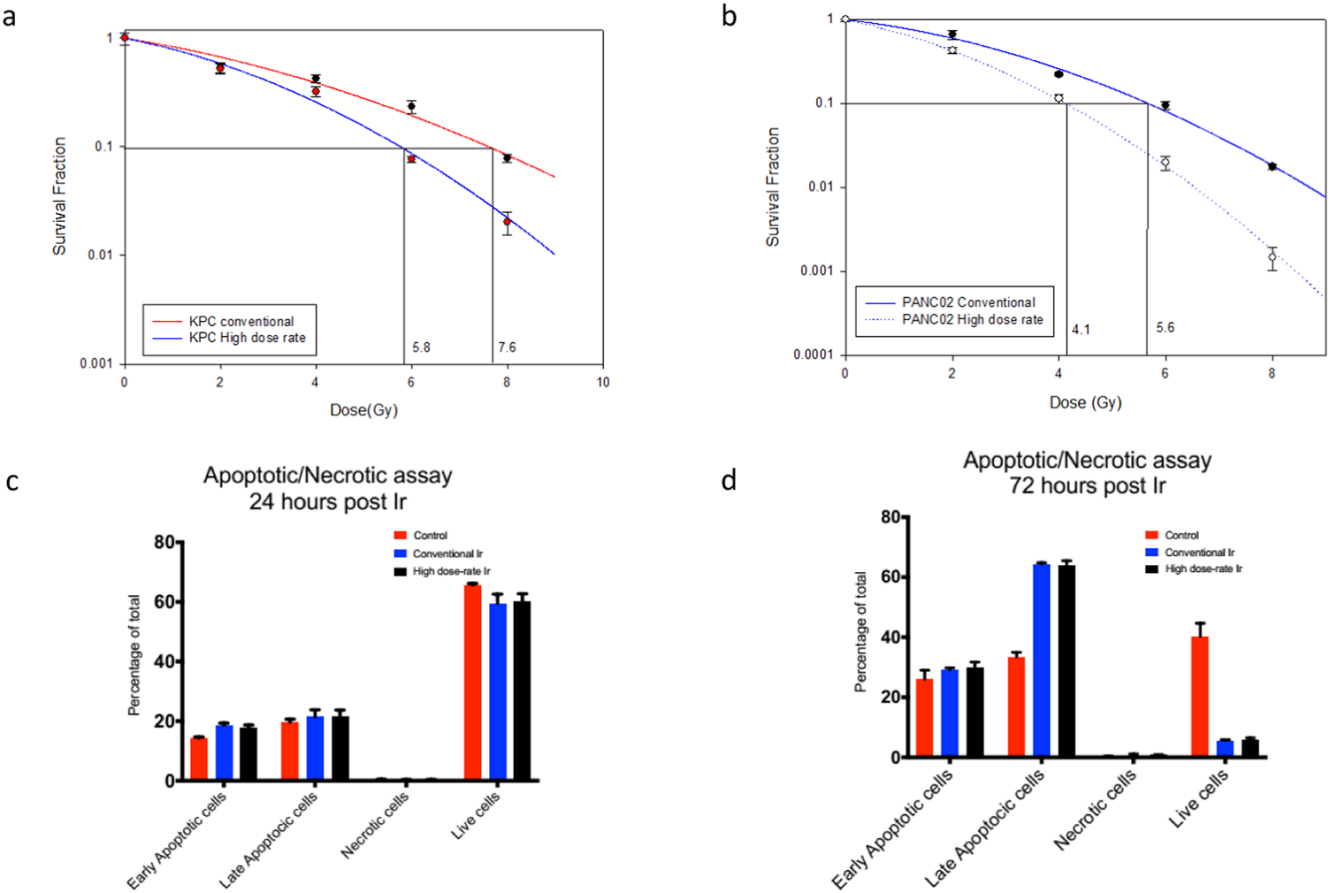
Ultra-high dose rate (35 Gy/s) RT is more potent than conventional dose rate (0.1 Gy/s) RT in killing tumor cells, with no appreciable difference in modes of cell death. (**a**) Clonogenic survival curve of KPC cells treated with ultra-high dose rate shows enhancement factor at 10% surviving fraction (DEF_10_) of 1.310 compared to conventional dose rate RT(0,2,4,6,8 Gy of radiation) (**b**) Clonogenic survival curve of Panc02 cells treated with ultra-high dose rate shows enhancement factor at 10% surviving fraction (DEF_10_) of 1.365 compared to conventional dose rate RT(0,2,4,6,8 Gy of radiation) (**c**,**d**) The percentage of PBMCs undergoing early apoptosis, late apoptosis, and necrosis at 24 h and 72 h following ultra-high and conventional dose rate RT. Dose enhancement ratio at survival fraction (DER_SF0.1_) of 10% was calculated by (radiation dose needed to kill 90% at high dose-rate)/(radiation dose needed to kill 90% with conventional dose rate). The radiation dose was calculated from the linear quadratic model based on the survival fraction at each dose. Data are derived from experiments conducted in sextuplicate.

We performed a classical clonogenic assay with KPC and Panc02 cell lines with conventional dose rate and ultra-high dose rate. The plating efficiency for KPC and Panc02 cells were 60%. DEF_10_ of KPC and Panc02 tumor cells were 1.310 and 1.365, respectively, as shown in Fig. 1a,b. These results suggest that ultra-high dose rate is more potent than conventional dose rate in reducing clonogenicity of cells.

### Ultra-high dose rate RT is as potent as conventional dose rate RT in killing lymphocytes *ex vivo*

To ascertain if ultra-high dose rate protects lymphocytes from radiation, we administered a single 2 Gy dose of radiation to PBMCs from healthy human subject’s *ex vivo* using both ultra-high and conventional dose rate RT. Using a kit that can simultaneously detect healthy, apoptotic, and necrotic cells^23^, we observed that ultra-high dose rate RT was as effective in killing PBMCs as conventional dose rate RT at 24 h and 72 h post-irradiation. There was no appreciable difference in mode of cell death between ultra-high and conventional dose rate RT, as shown in Fig. 1c,d. In both instances, cells death was predominantly via late apoptosis.

### Ultra-high dose rate RT does not spare the immune cells irradiated by cardiac irradiation

**Figure 2:**
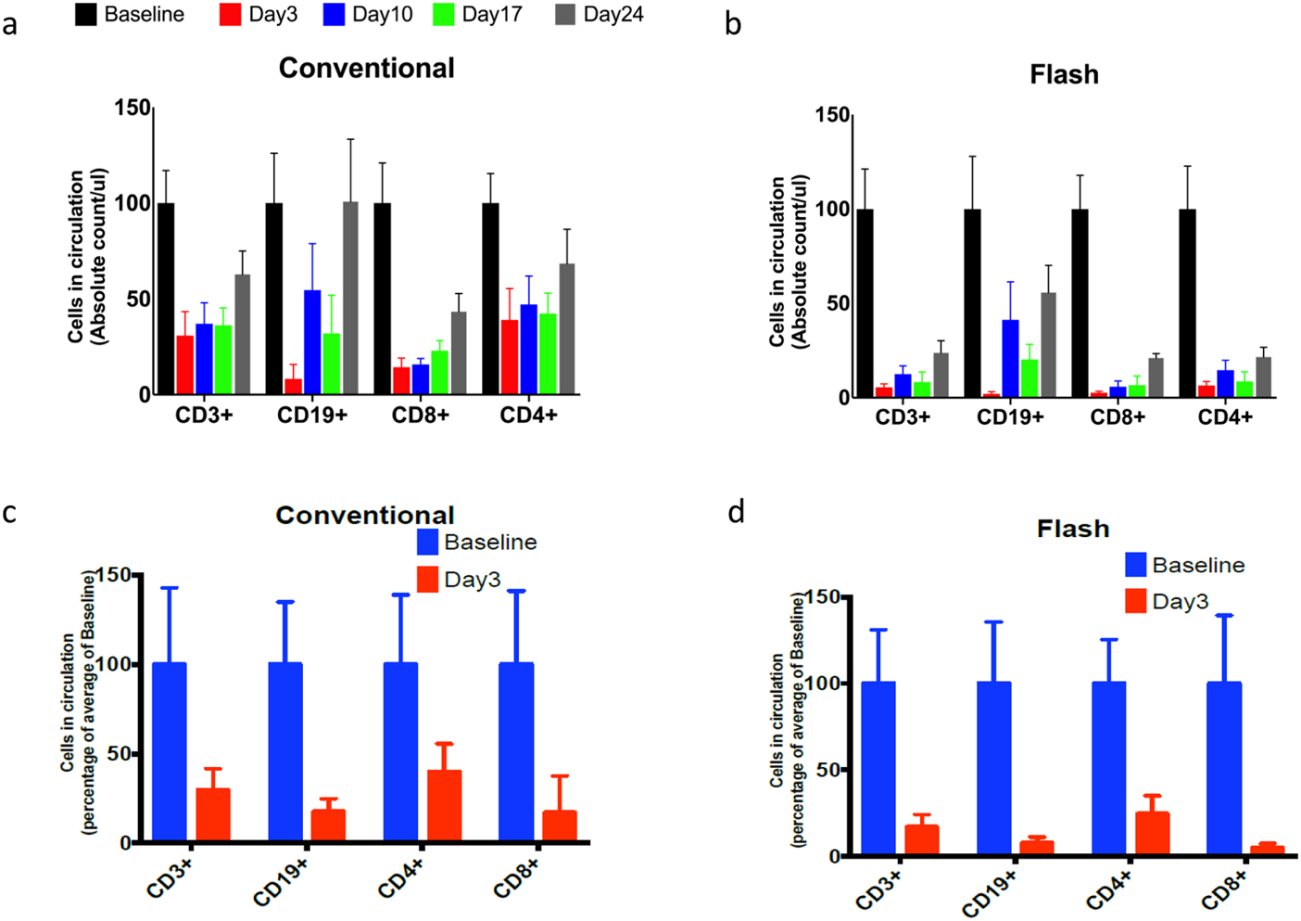
BALB/c mice undergoing cardiac irradiation with multi-fraction regimen of 2 Gy per day for 5 days or 10 Gy single fraction develop severe lymphopenia irrespective of dose rate. (**a**) Time trends of flow cytometric CD3, CD4, CD8, and CD19 lymphocyte counts in the peripheral blood of mice undergoing conventional dose rate RT with the multi-fraction regimen. (**b**) Time trends of flow cytometric CD3, CD4, CD8, and CD19 lymphocyte counts in the peripheral blood of mice undergoing ultra-high dose rate RT with the multi-fraction regimen. (**c**) Flow cytometric CD3, CD4, CD8, and CD19 counts in the peripheral blood of mice on day 3 following conventional dose rate RT with the single-fraction regimen of 10 Gy. (**d**) Flow cytometric CD3, CD4, CD8, and CD19 counts in the peripheral blood of mice on day 3 following ultra-high dose rate RT with the single-fraction regimen of 10 Gy. Data are the mean percentages of cells ± SE. *p < 0.05 compared between control and other cohorts. Data are derived from experiment conducted in triplicates. (n = 5 in control, high dose rate and conventional dose rate group).

We then compared the effect of conventional and ultra-high dose rate RT on circulating lymphocyte counts and subsets following cardiac RT *in vivo*. Female BALB/c mice were subjected to cardiac irradiation through a specially designed lead block and jig that irradiated just the heart with a 5 mm margin to a dose of 2 Gy for 5 consecutive days (cumulative dose of 10 Gy). The frequency and magnitude of depletion of circulating CD3, CD4, CD8, and CD19 cells was comparable between ultra-high dose rate RT and conventional dose rate RT. The lymphocyte depletion by ultra-high dose rate was more severe and sustained with ultra-high dose rate RT than conventional dose rate RT. On day 24 post-irradiation, CD3 cell counts recovered to 50% of baseline levels with ultra-high dose rate RT compared to 100% recovery for conventional dose rate RT. A similar pattern was observed for CD4, CD8, and CD19 cell populations as well, as shown in Fig. 2a,b. To assess if hypofractioanted RT with ultra-high dose rate can spare the lymphocytes, we evaluated a single 8 Gy fraction as well. Hypofractioanted ultra-high dose rate RT was more potent than conventional dose rate RT in depleting CD3, CD4, CD8, and CD19 cell populations, as shown in Fig. 2c,d. With the dose rates we used, we were unable to document any reduction in lymphopenia arising in the context of cardiac irradiation with ultra-high dose rate RT in either the multi-fraction setting or the single-fraction setting.

### Ultra-high dose rate RT does not spare immune cells irradiated by splenic irradiation

**Figure 3:**
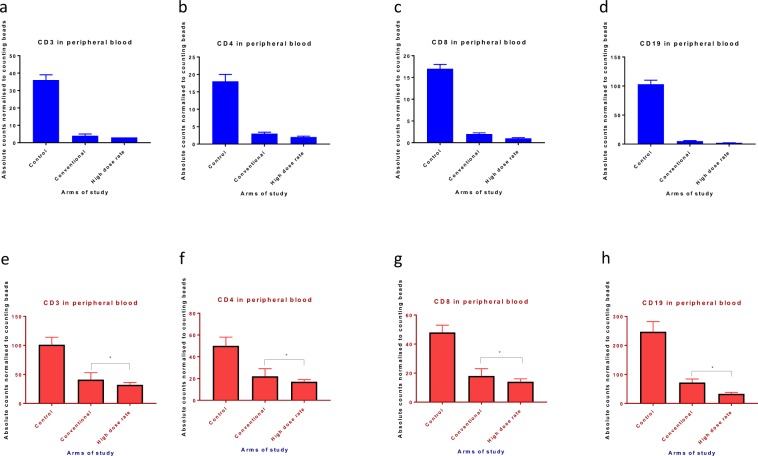
C57BL/6 mice undergoing splenic irradiation with a multi-fraction regimen of 1 Gy per day for 5 days or a single fraction of 5 Gy experience severe lymphopenia irrespective of dose rate. (**a**–**d**) Flow cytometric CD3, CD4, CD8, and CD19 counts in the peripheral blood of mice on day 3 following conventional or ultra-high dose rate RT with the multi-fraction regimen. (**e**–**h**) Flow cytometric CD3, CD4, CD8, and CD19 counts in the peripheral blood of mice on day 3 following conventional or ultra-high dose rate RT with a single fraction of 5 Gy. Data are the mean percentages of cells ± SE. *p < 0.05 compared between control and other cohorts. Data are derived from experiment conducted in triplicates. (n = 5 in control, high dose rate and conventional dose rate group).

We compared circulating lymphocyte levels following splenic irradiation with conventional and ultra-high dose rate RT. Male C57BL/6 mice were subjected to splenic irradiation through a specially designed lead block and jig that irradiated just the spleen with a 5 mm margin to a dose of 1 Gy daily for 5 consecutive days (cumulative dose of 5 Gy). As with cardiac irradiation, ultra-high dose rate RT depleted circulating CD3, CD4, CD8, and CD19 cells to a similar extent as conventional dose rate RT, as shown in Fig. 3a–d. As with cardiac irradiation, hypofractioanted (5 Gy single fraction) ultra-high dose rate RT was more potent than conventional dose rate RT in depleting the CD3, CD4, CD8, and CD19 cell populations as shown in Fig. 3e–h. Again, our results fail to demonstrate a prominent lymphocyte sparing effect with ultra-high dose rate splenic irradiation in either the multi-fraction setting or the single-fraction setting. If any, there was more lymphocyte depletion with ultra-high dose rate RT.

### Ultra-high dose rate RT is more potent in causing gastrointestinal mucosal toxicity than conventional dose rate RT

**Figure 4:**
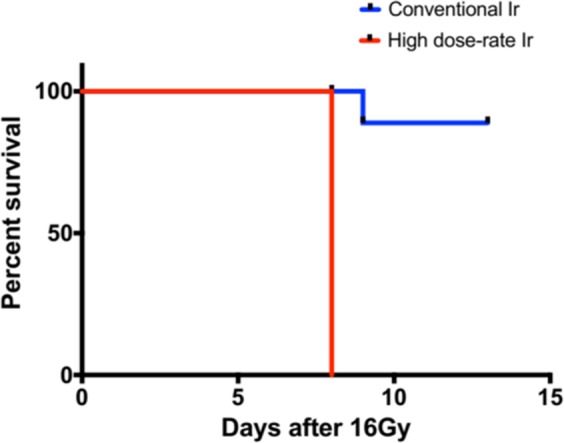
Ultra-high dose rate (35 Gy/s) RT is more potent than conventional dose rate (0.1 Gy/s) RT in inducing acute gastrointestinal syndrome after a single fraction of 16 Gy of whole abdominal radiation. (n = 5 in high dose rate and conventional dose rate group). The Kaplan Meier curve for the survival data, was determined by the log-rank test. Results with a *P* value of <0.05 were considered significant.

In a model of gastrointestinal mucosal injury from whole abdominal irradiation, BALB/c mice were exposed to a single fraction 16 Gy dose of RT administered with a specially designed lead block and jig. All mice treated with ultra-high dose rate RT died within 7 days whereas mice survived to day 15 after conventional dose rate RT to the abdomen, as shown in Fig. 4.

## Discussion

Our interest in FLASH RT stemmed from an attempt to find potential ways to prevent/reduce radiation-induced lymphopenia in esophageal, lung, and pancreatic cancer patients. Studies have suggested that the mean heart dose during esophageal and lung cancer RT and unintended splenic dose during pancreatic cancer RT contribute to RIL, which, in turn, results in inferior survival outcomes^24^. We hypothesized that ultra-high dose rates can spare the immune system while retaining tumoricidal activity. Favaudon *et al*. showed that RT doses ranging from 17 Gy to 30 Gy at a dose rate of ≥40 Gy/s with 4.5 MeV electrons significantly reduced the incidence of lung fibrosis^2^. Gruel *et al*. reported that a whole brain dose of 10 Gy with 4.5 and 6 MeV electrons at dose rates ranging from 0.1 Gy/s to 500 Gy/s resulted in greater memory sparing when the dose rate was greater than 100 Gy/s and a loss of neuroprotective effect when the dose rate was below 30 Gy/s^4^. Gruel *et al*. also reported a greater memory sparing effect of FLASH RT than conventional dose rate RT in rodents receiving a 10 Gy dose of whole brain RT with X-rays at a dose rate of 37 Gy/s^5^.

Our results demonstrate that ultra-high dose rates of 35 Gy/s fail to spare normal tissues to the extent observed in other reports using dose rates ranging from 30 Gy/s to 100 Gy/s. While these results may be specific to the models and assays we used and the dose rate we used, these results may highlight that normal tissue sparing with ultra-high dose rate RT is not universal and may depend on a number of additional, yet unknown, biological factors and/or treatment parameters. In particular, sparing of lymphocytes, one of the most radiosensitive cells that are ubiquitously present in all normal tissues and one of the key drivers of inflammatory responses to radiation, may not explain the wide therapeutic window reported for ultra-high dose rate RT. Similarly, ultra-high dose rate RT also failed to show a greater sparing effect on another relatively radiosensitive cell, the gastrointestinal mucosal epithelial cell. Comparing our results with that of others, there are a number of possible take-home messages. First, the dose rate effect may be tissue-specific with lymphocytes and gastrointestinal epithelial cells being cells with rapid cell turnover. Injury to these tissues could be repaired over time especially if sufficient numbers of stem cells survive and can repopulate the cell pools. On the other hand, neuronal damage and lung fibrosis are late effects occurring in slowly dividing tissues that are not fully repaired. While a component of lung fibrosis is potentially a consequential late effect, i.e., secondary to an acute effect (pneumonitis), it still is a late effect seen in a tissue without rapid cell turnover. Second, consistent with the notion of late responding tissue, with a low α/β ratio, being especially sensitive to dose per fraction the previously noted lung and brain injury sparing with ultra-high dose rate RT was especially prominent with a high dose/fraction (and total dose), 10 Gy for whole brain and 17 Gy or 30 Gy for lung fibrosis. This is less so with early responding tissue with a high α/β ratio where injury is more sensitive to treatment time or fractionation (and total dose). Third, viewed from the perspective of Casarett’s classification of radiation sensitivity, gastrointestinal epithelial cells span a spectrum from vegetative intermitotic (Group I), i.e., continually dividing without differentiating to differentiating intermitotic (Group II), i.e., differentiating into mature non-dividing cells after a finite number of divisions. Lymphocytes are considered a notable exception to the fixed post-mitotic group (Group IV) because they are exquisitely sensitive to RT (LD_50_, the lethal dose required to reduce the surviving lymphocyte population to 50% of initial values, is as low as 2 Gy) despite being permanently non-dividing^25^. Of particular note, heart and splenic irradiation do not deplete hematopoietic stem cells; these Group I cells are not depleted unless specifically targeted such as with marrow ablative RT regimens. On the other hand, lung alveolar cells (epithelial and stromal connective tissue cells) are likely reverting post-mitotic non-dividing cells (Group III) with the potential to revert to a dividing phenotype when needed. Brain neurons are classified as fixed post-mitotic (Group IV). In general, Groups I and II are are more radiosensitive than Groups III and IV. No doubt, these are generalizations and, indeed, different cells within an organ (e.g., neurons vs. glial cells vs. endothelial cells) differ in their response to radiation. Nonetheless, this difference in cell types between our studies and previous studies may explain some of the differing results we see. It could be that our studies targeted distinctly different types of normal tissues that behave differently in response to radiation. Fourth, the effects observed by different groups may be dependent on physical characteristics of the radiation beam, strain, sex, age at response, and post-irradiation evaluation time. Lastly, it is possible that our dose rates are not sufficiently high to observe some of the purported normal tissue sparing seen in other organs and other assays. Yet, they serve as a cautionary note for intuitively assuming that all ultra-high dose rate RT is normal tissue sparing. In fact, even while generating ultra-high dose rates at a deep-seated target like pancreatic cancer, upstream normal tissues along each radiation beam track may receive lower dose rates, not dissimilar to those that we utilized.

Although our results are contrary to what has been previously reported, they raise interesting questions about how ultra-high dose rate RT can have a normal tissue toxicity sparing effect based upon the organ irradiated, dose used, fraction size, beam energy, and the type of radiation being tested. The varied dose rates and endpoints that have been assessed in FLASH experiments have been summarized in Table 1. In addition, the optimal dose rate for FLASH radiation remains an unanswered question. From a translational research perspective, given the widespread availability of X-rays or protons, future studies should be geared towards employing these radiation beams in a fractionation and dosing format that is clinically relevant. Current configurations of cyclotron-based accelerators, more so that synchrotron-based accelerators, can more readily deliver the ultra-high dose rates needed for FLASH RT.

**Table 1:** Summarizes the dose rates, radiation type, radiation fraction size and the endpoints that have been assessed in different model systems that have assessed FLASH radiation as part of the experiments.

Author	Experiments	Model system	Type of radiation	Radiation fraction	Mean Dose rate	Comments
Favaudon *et al*.^2^	Mice	Lung fibrogenesis and blood vessels	4.5 Mev electrons	16 to 30 Gy of single fraction to bilateral thorax	≥40 Gy/sec	The study showed a complete lack of acute pneumonitis and late lung fibrosis after bilateral thorax irradiation of C57BL/6 J mice with FLASH.FLASH prevented both activation of the TGF-b/SMAD cascade and acute apoptosis in blood vessels and bronchi.
Loo *et al*. 2017(Abstract)^27^	Mice	GI syndrome	20 Mev electron	10 to 22 Gy single fraction to abdomen	>70 Gy/sec and >200 Gy/sec	Mice receiving 13–19 Gy, 29% survived 20 days after conventional vs. 90% after FLASH. LD50 of 14.7 Gy for conventional and 17.5 Gy for FLASH (16.6 Gy and 18.3 Gy for the 70 and 210 Gy/s cohorts, respectively)
Kim *et al*. 2017 (Abstract)^28^	Mice	Lung cancer model	NA	15 Gy single fraction to tumor	>50 Gy/sec	High dose Conventional radiation resulted in a rapid and reversible tumor vasculature collapse, which did not occur with high dose FLASH irradiation as determined by CD31 area densities, indicating that the biological effects differ between Conventional and FLASH.
Gruel *et al*.^4^	Mice	Brain cognition model	4.5 Mev and 6 Mev electron	10 Gy single fraction to whole brain	0.1 Gy/sec to 500 Gy/sec	Flash-RT neuroprotective effect is lost below 30 Gy/s but fully preserved above 100 Gy/s
Vozenin *et al*.^3^	Mini-pigs and cat	Skin	4.5 Mev and 6 Mev electron	25–41 Gy single fraction to normal skin and skin tumors	300 Gy/sec	Single dose FLASH-RT shows promise as a new treatment option for cat patients with locally-advanced squamous cell carcinoma of the nasal planum. Our results in pig and cats provide a strong rational for further evaluating FLASH-RT in human patients
Gruel *et al*.^5^	Mice	Brain cognition model	X-rad 225 photons	10 Gy single fraction to brain	37 Gy/sec	Preservation of memory at two and six months after a 10 Gy single dose FLASH-X-rays WBI delivered at a mean dose-rate of 37 Gy/s
Beyreuther *et al*.^29^	Zebra embryo fish	Embryonic survival, rate of pericardial edema and, rate of spinal curvature	224 Mev protons	0 to 42.5GY of single fraction	100 Gy/sec	Significant protective effect of proton Flash could be revealed neither for the survival nor for the morphological integrity of the zebrafish embryos. Solely for the rate of pericardial edema, a significantly reduced effect was found at the 3rd and 4th day after 23 Gy proton Flash compared to conventional proton irradiation
Buonanno *et al*.^30^	Human lung fibroblast cells	Clonogenic assay, DNA damage and senescence	4.5 Mev protons	0,5,20 Gy	0.05, 100 or 1000 Gy/s	To characterize the clonogenic cell survival depending on the proton dose rate, cells were exposed to different doses delivered at 0.05, 100 or 1000 Gy/s. The survival curves for all three dose rates followed a typical exponential decay trend with the dose. Although a slight difference between the low (0.05 Gy/s) and the two FALSH dose rates (100 and 1000 Gy/s) can be observed at the highest dose tested (10 Gy) the trends were not statistically different
Bourhis *et al*.^31^	Patient	Skin tumor	5.6 Mev electrons	15 Gy single fraction		First FLASH-RT treatment was feasible and safe with a favorable outcome both on normal skin and the tumor
Gruel *et al*.^32^	Mice	Brain cognition model	6 Mev electrons	10 to 14 Gy of single fraction to whole brain	>100 Gy/sec	FLASH did not cause radiation-induced deficits in learning and memory in mice, did not impair extinction memory. FLASH produced lower levels of the toxic reactive oxygen species hydrogen peroxide, did not induce neuroinflammation
Simmons *et al*.^33^	Mice	Brain cognition model	16 or 20 Mev electrons	30 Gy single fraction whole brain	300 Gy/sec for 16 Mev or 200 Gy/sec for 20 Mev	FLASH is associated with reduced cognitive deficits, less loss of hippocampal dendritic spines

In conclusion, our study shows that at dose rates of 35 Gy/s there is no immune compartment sparing in cardiac and splenic models of lymphopenia or gastrointestinal mucosal injury. The optimal dose rate for sparing these normal tissues, if any, remains to be defined. Future experiments should be geared towards defining the optimal dose, dose rate, and fraction size for reducing specific normal tissue complication probabilities for specific organs irradiated in protocols that mimic clinical treatment scenarios.

## Materials and Methods

### Cell lines and reagents

The murine pancreatic cancer cell line, KPC, was obtained from the American Type Culture Collection (Manassas, VA). The murine pancreatic cancer cell line, Panc02, was obtained from the Characterized Cell Line Core Facility at MD Anderson Cancer Center. KPC and Panc02 were cultured under sterile conditions and with media recommended by the supplier. Human peripheral blood mononuclear cells (PBMCs) from healthy subjects was purchased from Stem Cell Technologies (stemcell.com). All reagents were of analytical grade.

### Irradiation device and dosimetry

We disassembled the gantry head of a decommissioned Varian 2100 IX linear accelerator to deliver 20 MeV electrons at a nominal dose rate of 35 Gy/s at the level of the mirror within the collimator, similar to the approach described by Maxim *et al*.^26^. This entailed use of the gun current settings for 6 MV photons, removal of the target and the flattening filter, and rigging of the gating relay to start and stop irradiation within milliseconds under automatic control. We confirmed dose, dose rate and field uniformity using EBT3 film, thermoluminescent dosimeters (TLDs), Farmer ion chambers, and a parallel plate chamber. EBT3 film and a Farmer ion chamber measurement were used for each experimental irradiation. For the conventional irradiation, we used a Varian True Beam and delivered radiation at 0.1 Gy/sec. Mice were irradiated in either our modified decommissioned linear accelerator which was tuned to deliver ultra-high dose rates of 20 Mev electrons on the order of 35 Gy/sec or with our conventional True Beam linear accelerator which delivered dose at 0.1 Gy/sec. In our ultra-high dose rate FLASH irradiation system, the mice were irradiated on a platform in the head of the linear accelerators gantry with customized lead cutouts that focused the radiation only on the areas of interest. The lead cutouts were either 2 cm × 2 cm or 4 cm × 4 cm. See Supplementary data.

### Clonogenic assay

Panc02 and KPC were seeded in 30 mm Petri dishes at varying concentrations and incubated overnight. The dishes were treated with 0, 2, 4, 6, and 8 Gy of conventional dose rate and ultra-high dose rate RT. The dishes were left undisturbed for around 8 days at 37 °C in a 5% CO_2_ incubator until colonies formed. When colonies became visible to the naked eye, the plates were stained with 0.5% crystal violet diluted in 95% ethanol, and photographed at high resolution. Supervised automated counting with an Oxford optotronix gel counter enumerated the number of surviving colonies (>50 cells per colony). This surviving colony fraction was plotted against radiation dose, and dose enhancement factors at 10% surviving fraction (DEF_10_), defined as the ratio of dose that results in 10% surviving fraction at conventional dose rate to dose that results in 10% surviving fraction at ultra-high dose rate, was calculated using Sigma plot. Each experiment was done in triplicate.

### Animal experiments

All animal procedures were conducted according to the Guide for the Care and Use of Laboratory Animals, prepared by the Institute of Laboratory Animal Resources, National Research Council and National Academy of Sciences, and the MD Anderson Cancer Center Institutional Animal Care and Use Committee. The experimental protocols were approved by the Institutional Animal Care and Use Committee. C57BL/6 male mice were purchased from Experimental Radiation Oncology Animal Facility at MD Anderson and BALB/c female mice were purchased from Charles River laboratory and housed in an air-conditioned facility accredited by the Association for Assessment and Accreditation of Laboratory Animal Care International. Mice were housed (no more than 5 per cage) in individually ventilated Tecniplast cages in a room with a HEPA-filtered air supply at relative humidity of 60% ± 10% on a 12-hour light/dark cycle. All mice were quarantined for 3 days before any experiments were begun. Food (Purina PicoLab Rodent Diet 5053, Harlan Teklad, WI, USA) and water (reverse osmosis chlorinated or acidified water, pH 2.5–2.8) were provided ad libitum.

### Flow cytometry

Facial venous blood was collected for peripheral blood flow cytometry. The peripheral blood was obtained in a heparin coated tube and ACK lysis was performed for 20 mins. The lymphocytes were washed twice in phosphate buffered saline and then incubated on ice for 45 mins with a cocktail of primary antibodies for CD45, CD3, CD4, CD8, and CD19, with Zombie aqua used for live/dead discrimination. Then, cells were washed and fixed with 1.6% paraformaldehyde. The cells were run through a Gallios 561 flow cytometer and the collected data was analyzed using Kaluza software.

### Apoptosis and necrosis quantification

Peripheral blood mononuclear cells (PBMCs) from healthy human subjects was acquired from Stem Cell Technologies (Vancouver, Canada). One million PBMCs were irradiated with single 2 Gy radiation doses (ultra-high and conventional dose rates). Apoptosis and necrosis were quantified after 24 h and 72 h by flow cytometry using a kit which can simultaneously detect apoptotic, necrotic, and healthy cells as per the manufacturer’s protocol (Biotium,Inc., Hayward, USA).

### Statistical analysis

All experiments were carried out in triplicate otherwise specified. Results are presented as means and standard errors (SE). Statistically significant differences were calculated by using two-tailed unpaired *t* tests or by one-way analysis of variance. The Kaplan Meier curve for the survival data, was determined by the log-rank test. Results with a *P* value of < 0.05 were considered significant.

## Supplementary information

Supplementary information

## Data Availability

The datasets generated during and/or analyzed during the current study are available from the corresponding author on reasonable request.
