# Author Correction: Ultra high dose rate (35 Gy/sec) radiation does not spare the normal tissue in cardiac and splenic models of lymphopenia and gastrointestinal syndrome

**DOI:** 10.1038/s41598-020-67913-7

**Published:** 2020-06-30

**Authors:** Bhanu Prasad Venkatesulu, Amrish Sharma, Julianne M. Pollard-Larkin, Ramaswamy Sadagopan, Jessica Symons, Shinya Neri, Pankaj K. Singh, Ramesh Tailor, Steven H. Lin, Sunil Krishnan

**Affiliations:** 1 Departments of Experimental Radiation Oncology, University of Texas MD Anderson Cancer Center, Houston, TX USA; 2 Radiation Oncology, University of Texas MD Anderson Cancer Center, Houston, TX USA; 3 Department of Radiation Physics, University of Texas MD Anderson Cancer Center, Houston, TX USA; 4 The University of Texas MD Anderson Cancer Center-UT Health Graduate School of Biomedical Sciences, Houston, TX USA; 5 Department of Radiation Oncology, Mayo Clinic Florida, 4500 San Pablo Road S, Jacksonville, FL 32224 USA

Correction to: *Scientific Reports* 10.1038/s41598-019-53562-y, published online 20 November 2019

In this Article, details on beam pulse structure, geometry, beam flatness and inferred instantaneous dose rate were omitted; as a result, the Materials and Methods should contain the following section:

“**Beam Parameters**

We used a pulse rate of 180Hz, pulse length of 4us, 20 MeV electrons, and a uniform dose/dose rate over a 2 × 2 cm^2^ or 4 × 4 cm^2^ field. Figure 1 below illustrates flatness and dose uniformity of these fields. The in-plane (A) and cross-plane (B) 80% widths for the 2 × 2 cm^2^ cut-out were both 1.8 cm whereas that for the 4 × 4 cm^2^ cut-out were 4.0 cm and 2.9 cm, respectively.

**Figure 1:**
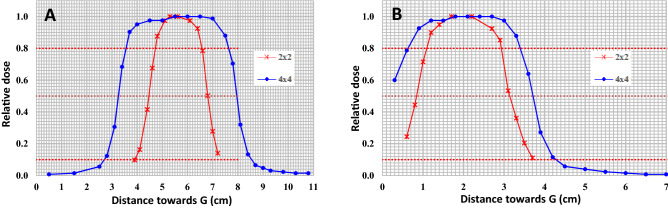
EBT3 film dosimetry of 2 × 2 cm and 4 × 4 cm fields at 26.2 cm source-to-skin-distance demonstrating flatness of the fields in the radial (**A**) and transverse (**B**) planes of a 20 MeV electron beam output from a clinical linear accelerator.

The dose rates were calculated by dividing the dose measured using film by the gate time set for the delivery. The dose rates ranged from 66% to 115% of average dose rate of 32.6 Gy/s for the 2 × 2 cm^2^ field. The corresponding values for the open 4 × 4 cm^2^ field were 96% to 131% of the average dose rate of 38.8 Gy/s. A larger uncertainty in dose rates when delivering small doses over short gate times is likely due to a greater contribution by timer error. This initial delay or timer error of about 31 ms is estimated by the x-intercept of the calibration curve, Fig. 2 below, which plots the charge collected using an A16 ion-chamber (in nC) against gating signal width (in ms). Although the delivered dose rate was linear with time, this initial delay has a greater impact on irradiation time for smaller doses.

**Figure 2:**
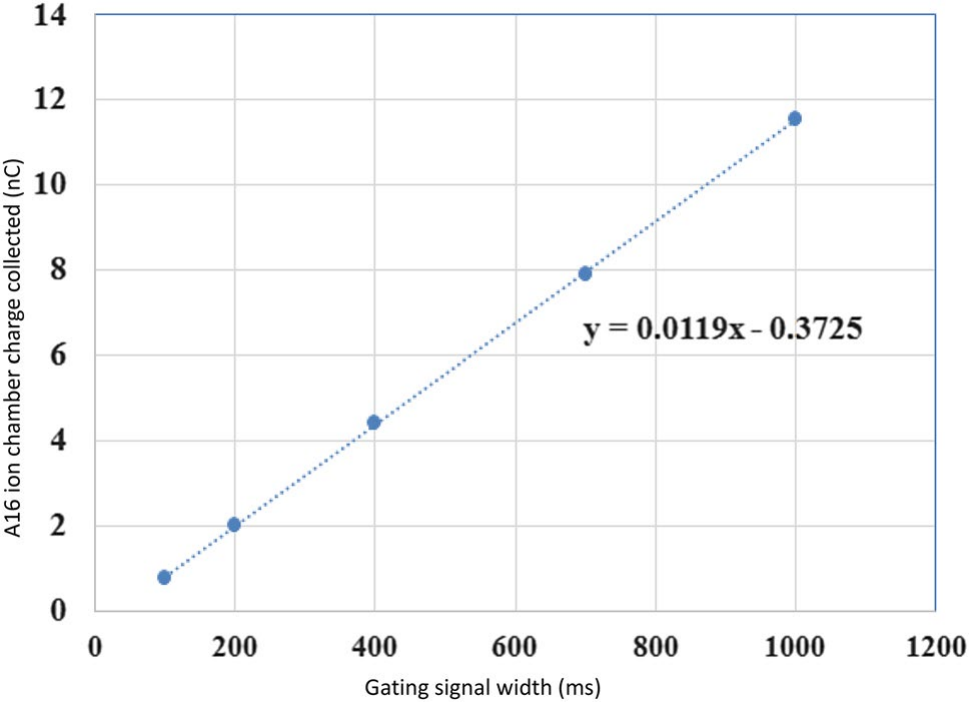
Calibration curve of dose delivered as a function of gating signal width.

Instantaneous dose rate measurements require sophisticated controlled per-pulse delivery of the radiation by use of an oscilloscope programmed with an advanced gating control system and a dosimeter providing real time measurements. At the time of our experiments, since instantaneous dose rate was not shown to be critical for the observed FLASH effect, we did not initially set up our system to measure dose per pulse. Towards the end of our experiments (before the linear accelerator was abandoned and removed), we attempted to measure instantaneous dose rate with our Arduino and oscilloscope setup, but were not able to get the beam to terminate precisely with each pulse to make an accurate measurement. While directly measuring the extremely high dose per pulse or instantaneous dose rate was outside of our ability, we were able to directly measure average dose rate for several experimental setups in ultra-high dose rate mode utilizing calibrated ionization chambers and EBT 3 film and via independent verification by a third party, Radiation Dosimetry Services (RDS). Our agreement with the RDS verification ranged from 0% for 8 Gy × 2 irradiations to within 5% for 2 Gy × 6 irradiations. The Arduino board used to time the dose delivery system had its timing calibration verified using an oscilloscope. The dose-rate servo-control and the monitor chamber system were by-passed. Lastly, the reported values are based on measurements using time not pulse to deliver dose. In the text therefore, an estimate based on the nominal 180 pulses/sec (as quoted by Varian for standard operating configuration) was provided.”

The authors were not explicit in the distinction between the present work and previous literature, and therefore, the following text in the Discussion,

“The optimal dose rate for sparing these normal tissues, if any, remains to be defined.”

should read:

“The optimal dose rate for sparing these normal tissues, if any, remains to be defined. It is worth noting that the primary motivation for our manuscript was to determine whether lymphocyte sparing by use of ultra-high dose rate radiation might result in normal tissue sparing effects, and not necessarily to recreate FLASH exactly as described by previous groups, which has been rather inconsistent and variable. Our results therefore do not dispute the findings of other groups but merely serve as a cautionary note for future researchers alerting them to our inability to see a normal tissue sparing effect in our experiment – defined strictly by our beam geometry and our narrowly defined biological endpoints.”

