# Supplementary material for: Ultra high dose rate (35 Gy/sec) radiation does not spare the normal tissue in cardiac and splenic models of lymphopenia and gastrointestinal syndrome
Source: Sci Rep. 2019 Nov 20;9:17180. doi: 10.1038/s41598-019-53562-y (PMC6868225; doi:10.1038/s41598-019-53562-y)
Supplement: Supplementary file 1 — Supplementary information [file 41598_2019_53562_MOESM1_ESM.pdf]

Ultra high dose rate (35Gy/sec) radiation does not spare the normal tissue in cardiac and splenic models of lymphopenia and gastrointestinal syndrome

Bhanu Prasad Venkatesulu[1a] MD, Amrish Sharma[1a] PhD, Julianne M. Pollard-Larkin[3] PhD, Ramaswamy Sadagopan[3] MS, Jessica Symons[1, 4] BS, Shinya Neri[1] MD PhD, Pankaj K Singh[1] PhD, Ramesh Tailor[3] PhD, Steven Hsesheng Lin[1,2,4, *] MD PhD, Sunil Krishnan[1, 2, 4, *] MD

Departments of Experimental Radiation Oncology[1] and Radiation Oncology[2], Department of Radiation Physics[3] University of Texas MD Anderson Cancer Center, Houston, Texas, and The University of Texas MD Anderson Cancer Center-UT Health Graduate School of Biomedical Sciences[4], Houston, Texas, USA

*Corresponding authors: Sunil Krishnan, MD, FACP, FASTRO, Department of Radiation Oncology, Unit 097, Y6.6006a, The University of Texas MD Anderson Cancer Center, 1515 Holcombe Blvd. Houston, TX USA 77030. Tel 713-563-2377; Fax 713-745-2186; E-mail: krishnan.sunil@mayo.edu; Steven H. Lin MD PhD, The University of Texas MD Anderson Cancer Center, 1515 Holcombe Blvd., Houston, TX 77030. Phone: 713-563-8490; Fax: 713-563-2366; E-mail: shlin@mdanderson.org.

[a] These authors contributed equally to the manuscript.

Supplementary Figure legends:

Figure 1

(A) The experimental ultra-high dose-rate linear accelerator. (B) The experimental set-up for splenic irradiation with ultra-high dose rate RT wherein a lead shield is placed just above level of the mirror inside the gantry head and a 2 cm aperture directs the beam to the spleen of mice. (C) The experimental set-up for splenic irradiation with conventional dose rate RT wherein a lead shield is placed in the electron cone head and a 2 cm aperture directs the beam to the spleen of mice. (D) The experimental set-up for cardiac irradiation with ultra-high dose rate RT wherein a lead shield is placed just above level of the mirror inside the gantry head and a 2 cm aperture directs the radiation to the heart of mice. (E) The experimental set-up for cardiac irradiation with conventional dose rate RT wherein a lead shield is placed in the electron cone head and a 2 cm aperture directs the radiation to the heart of mice.

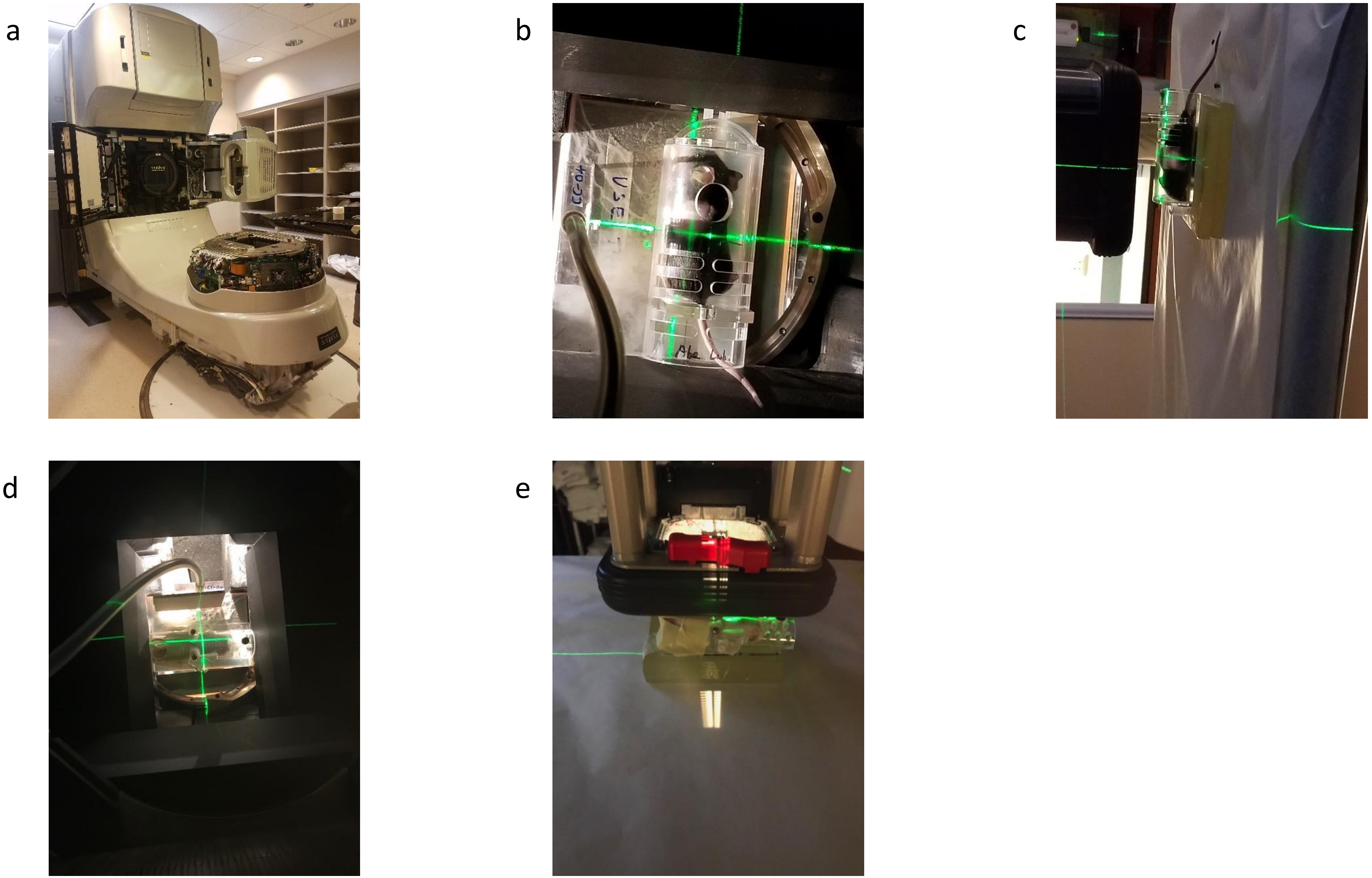
a
b
c
d
e
